# Side effects associated with the use of dexamethasone for prophylaxis of delayed emesis after moderately emetogenic chemotherapy

**DOI:** 10.1038/sj.bjc.6603048

**Published:** 2006-03-21

**Authors:** J Vardy, K S Chiew, J Galica, G R Pond, I F Tannock

**Affiliations:** 1 Department of Medical Oncology and Hematology, Princess Margaret Hospital, University of Toronto, 610 University Ave, Toronto, Ontario, Canada M5G2M9

**Keywords:** dexamethasone, side effects, nausea, vomiting, chemotherapy

## Abstract

The role of dexamethasone to reduce delayed emesis following highly emetogenic chemotherapy is proven, but there is less evidence of benefit after mild–moderately emetogenic regimens. Here, we develop and evaluate a Dexamethasone Symptom Questionnaire (DSQ) to assess the side effects of dexamethasone in the week after patients receive moderately emetogenic chemotherapy. The DSQ was first optimised with the aid of a focus group. Sixty patients receiving oral dexamethasone for prophylaxis of delayed emesis after moderately emetogenic chemotherapy for cancer completed and then evaluated the DSQ. Patients reported that the DSQ was clearly worded and addressed items important to them. Patients receiving dexamethasone reported moderate–severe problems with insomnia (45%), indigestion/epigastric discomfort (27%), agitation (27%), increased appetite (19%), weight gain (16%) and acne (15%) in the week following chemotherapy. The side effects of dexamethasone may outweigh its benefits when used with moderately emetogenic chemotherapy. A randomised, double-blind crossover trial is underway to determine the effect of dexamethasone on nausea and vomiting, and the impact of side effects of dexamethasone and of nausea and vomiting on quality of life.

Dexamethasone given before chemotherapy has been shown to reduce acute nausea and vomiting (Cassileth *et al*, 1983; Kris *et al*, 1989; Smith *et al*, 1991; Smyth *et al*, 1991; Roila *et al*, 1991, 1992; Hesketh *et al*, 1994, 1995; Adams *et al*, 1995; Latreille *et al*, 1995; Lofters *et al*, 1997; Warr, 1997; Ioannidis *et al*, 2000). Dexamethasone given after highly emetogenic chemotherapy reduces delayed emesis (1997a, 1997b; Herrstedt *et al*, 1998; Ioannidis *et al*, 2000) and is recommended by all the antiemetic guidelines (1997a, 1997b, 1999; Gralla *et al*, 1999, 2001; Koeller *et al*, 2002). However, dexamethasone has side effects and there is less evidence for its benefit when given after mild–moderately emetogenic regimens (Roila *et al*, 1996, 2005; Ballatori *et al*, 1997).

Many patients complain of side effects associated with the use of dexamethasone despite it being reported as ‘well tolerated’ in antiemetic studies (Aapro *et al*, 1984). However, the side effects of dexamethasone are not evaluated specifically in most antiemetic studies, possibly because it is difficult to distinguish them from the effects of the chemotherapy, of concomitant antiemetics or of the intravenous dexamethasone given for control of acute emesis before chemotherapy.

Here we report the development and initial evaluation of the Dexamethasone Symptom Questionnaire (DSQ), a 13-item self-report questionnaire designed to measure the incidence and severity of symptoms and signs that may be caused by dexamethasone in the week following treatment.

Our aim was to optimise and validate the DSQ, and to provide pilot information about the incidence and severity of side effects that may be due to oral dexamethasone when used for delayed antiemetic prophylaxis after moderately emetogenic chemotherapy in cancer patients.

## MATERIALS AND METHODS

The DSQ is a self-report questionnaire designed to be completed 1 week after moderately emetogenic chemotherapy, and patients are asked to rate the incidence and severity of any of the side effects within the preceding 7 days. Using the framework of Kirshner and Guyatt (1985), it is intended as a descriptive measurement (to determine incidence and severity of side effects), as well as evaluative (to examine changes in side effects due to dexamethasone between cycles of chemotherapy).

### Development of the DSQ

The authors performed item generation based on their own clinical experience and after literature review and informal consultation with medical oncologists and non-physicians involved in cancer care. A questionnaire containing 13 questions was generated and responses formatted using a four-point Likert scale (1=not at all, 2=a little bit, 3=quite a bit, 4=very much) (Edwards, 1983).

Two small focus groups of patients, who were receiving moderately emetogenic chemotherapy and dexamethasone for delayed emetic prophylaxis, were conducted by facilitators (JV and KC). Patients completed the DSQ individually and then the group discussed each individual item, focusing on whether the items were important to the patients, and whether additional items should be included; they also commented on the clarity and wording of the questions and the options for response.

### Evaluation of side effects of dexamethasone

Following its optimisation, the DSQ was administered as a self-report questionnaire on one occasion, 1 week after chemotherapy, to a total of 60 patients. At the completion of the questionnaire, all patients were asked to provide a brief written evaluation of the DSQ. They were asked to comment on whether the questions were clear and easily understandable, whether they had experienced any other symptoms that were not listed and to suggest improvements to the questionnaire.

Demographic information was collected from the patient, and details of antiemetics and chemotherapy were collated from the medical records. Eligibility criteria for the patients were >18 years of age and receiving oral dexamethasone after moderately emetogenic chemotherapy for cancer as an outpatient at Princess Margaret Hospital. Patients with a major pre-existing psychiatric history, drug or alcohol abuse, severe co-morbidity or patients receiving steroids for indications other than for emetic control were excluded. Approval for the trial was obtained from the hospital Research Ethics Board, and informed consent was obtained from all participating patients.

### Scoring of the DSQ

The questionnaire comprises two constructs, which are scored separately with each item being scored from 0 to 3 (i.e. not at all=0 points; 2 a little bit=1 point, etc.). The main construct includes nine items that evaluate the following side effects of dexamethasone: insomnia, gastro-oesphageal reflux, agitation, increased appetite, weight gain, acne, hiccups, oral candida and depression on ceasing medication. Scores for this construct can range from 0 to 27. The second construct includes two items that evaluate the severity of nausea and vomiting (scores range from 0 to 6). A higher score indicates worse symptoms. Weight loss and decreased appetite are not included in either subscore but can be used as supportive information. Each individual item can also be scored independently to indicate the severity of the symptom within a group of patients.

### Statistical analysis

Demographic variables, treatment and side effects are presented as frequency data with number and percentage, together with 95% confidence intervals for the side effects. The Spearman rank coefficient was used to evaluate correlation between items of the DSQ. The remainder of the data is presented using descriptive statistics.

## RESULTS

### Development of the DSQ

The original questionnaire contained 13 items. Item reduction was performed using a clinimetric approach (Feinstein, 1983). After the focus groups and expert review (by three medical oncologists and an experienced cancer nurse), a question related to indigestion was judged to be redundant and was deleted, and an item related to depression was added. Minor adjustments in wording were made to two questions. The final version of the DSQ is shown in Table 1. Patients in the focus groups reported that the DSQ addressed all the items important to them and that the questions were clear and concise.

### Patient characteristics

Sixty cancer patients receiving moderately emetogenic chemotherapy (as defined by the Perugia guidelines (1998)) completed the DSQ 1 week after chemotherapy. There were 46 female and 14 male patients aged 28–78 years (median and mean 53 years). Before chemotherapy, all patients received dexamethasone (98% intravenously (10 or 20 mg) and one patient orally) and 90% had i.v. granisetron 1 mg. All patients received oral dexamethasone for delayed prophylaxis (35 received 4 mg twice daily for 2 days, 13 received 4 mg twice daily for 3 days, seven received 4 mg twice daily for 1 day and the remainder received 4 mg b.i.d. for 1–2 days and then a tapering dose). A total of 80% were also prescribed oral granisetron for delayed prophylaxis (generally 2 mg daily for 2 days) and a minority took prochlorperazine (35%), domperidone (15%) or metoclopramide (3%), as required for nausea or vomiting.

The tumour sites and chemotherapy regimens are listed in Table 2. The majority of the patients had breast, colorectal or ovarian cancer, and 50% were being treated for metastatic disease. The most common regimens were cyclophosphamide, epirubicin and 5-fluorouracil (CEF); adriamycin and cyclophosphamide (AC)±followed by a taxane; and oxaliplatin, 5-fluorouracil and folinic acid (FOLFOX). The mean number of chemotherapy cycles completed was four and there was a mixture of chemotherapy naïve and pretreated patients (Eight patients were receiving their first cycle of chemotherapy, 17 cycle 2, 10 cycle 3, 5 cycle 4 and the remainder had received five or more cycles of chemotherapy).

### Side effects associated with dexamethasone

The percentage of patients experiencing each of the symptoms, in the week following moderately emetogenic chemotherapy, and their self-reported severity rating according to the DSQ are shown in Table 3. Patients receiving dexamethasone reported moderate–severe problems with: insomnia (45% (95% CI 32–58%)), indigestion/epigastric discomfort (27% (95% CI 16–40%)), agitation: (27% (95% CI 16–40%)), increased appetite (19% (95% CI 10–30%)), weight gain (16% (95% CI 8–29%)) and acne (15% (95% CI 7–27%)), in the week following their chemotherapy. Only 15 patients (25%, 95% CI 15–38%) reported no or only mild side effects on all items, while 19 (32%, 95% CI 20–45%) reported at least three moderate–severe symptoms. Sixteen patients (27%, 95% CI 16–40%) reported moderate–severe symptoms for at least two of the following four: insomnia, gastro-oesphageal reflux disease, agitation and depression; only 21 patients (35%, 95% CI 23–48%) did not report moderate–severe symptoms for this cluster of side effects.

Moderate–severe vomiting was reported by five patients (8%, 95% CI 3–18%) and nausea by 17 patients (28%, 95% CI 17–41%). Eleven patients (18%, 95% CI 10–30%) scored ⩾3/6 on the emetic subscale, which included five patients (8%, 95% CI 3–18%) scoring 4 or above.

The only missing data were single items in two of the 60 questionnaires (one for hiccups and one for agitation), for which the symptom response was conservatively assigned to be ‘not at all.’ Five additional patients completed the questionnaire, but as they had not received oral dexamethasone after their chemotherapy, they were ineligible and excluded from analysis.

### Correlation between items of the DSQ

In order for the DSQ to be a useful clinical instrument, it should demonstrate correlation between items that one would expect to be correlated, and to discriminate between items that are expected to be relatively independent. Table 4 indicates Spearman rank correlation co-efficients between items of the DSQ. As expected, nausea and vomiting (Spearman *ρ*=0.38, *P*-value=0.003), and increased appetite and weight gain (Spearman *ρ*=0.50, *P*-value<0.001) were strongly correlated. Items such as insomnia and acne showed, as expected, a low coefficient of correlation (Spearman *ρ*=0.10, *P*-value=0.47). The nausea/vomiting subscore was positively associated with the dexamethasone side-effect subscore (Spearman *ρ*=0.17); however, this association was not statistically significant (*P*-value=0.19).

## DISCUSSION

The number of patients with moderate–severe symptoms that may be due to dexamethasone is substantial, with approximately half reporting insomnia and a quarter reporting each of gastro-oesophageal reflux and agitation of moderate–severe severity. Many patients experienced multiple symptoms. These results suggest that dexamethasone may have a substantial deleterious effect on quality of life (QOL).

Most patients receiving moderately emetogenic chemotherapy have well-controlled emesis: for example, (Gralla, 1997) reported rates of complete control of acute emesis in 90% and delayed emesis in 80–95%. In this study, 8% of patients reported moderate–severe vomiting and 28% reported moderate–severe nausea. Vomiting and nausea can decrease QOL, but QOL depends on multiple additional factors. Osoba (1997), Osoba *et al* (1997) and Rusthoven *et al* (1998) found a significant decline in several domains of QOL following chemotherapy in patients who reported no nausea and vomiting, although to a lesser extent than in patients with nausea and/or vomiting. The QOL of patients with minimal nausea or vomiting might be affected more by the side effects of the antiemetic treatment.

The DSQ has good face and content validity and sensibility. The purpose and framework is clearly delineated, and it has been tested in the population for which it is ultimately intended. The burden on respondents is low, with the questionnaire taking 2–3 min to complete. Patients and experts report that it is simple yet comprehensive. Further evaluation of construct validity and of test–retest reliability is required for full validation. Repeated measures will be evaluated in our future study.

The major limitation of the current study is that it is not possible to determine which side effects were caused by the dexamethasone given after chemotherapy, by the intravenous dexamethasone given before chemotherapy, by the chemotherapy itself, or by other antiemetics or concomitant medications. However, the symptoms are characteristic of those due to dexamethasone (and not of other drugs received by the patients).

The purpose of this pilot study was to determine whether sufficient patients suffer side effects from oral dexamethasone to warrant performing a definitive trial of its use. Because of the high rate of reported symptoms, a randomised double-masked placebo-controlled crossover trial is now underway to compare the use of dexamethasone with placebo for delayed emetic prophylaxis following moderately emetogenic chemotherapy. The trial will evaluate the impact of both dexamethasone and of nausea and vomiting on QOL and determine patients’ preference for either dexamethasone or placebo. In this trial, the two items regarding weight gain and weight loss will be omitted from the DSQ, as patients will be weighed to gain more accurate information on changes in weight. We will also omit the ‘decreased appetite’ item. The nausea and vomiting items could also be omitted in future studies if daily emetic dairies or more comprehensive emetic questionnaires were being used as part of an antiemetic study, and only information on the dexamethasone side effects was required.

The DSQ is a simple, brief instrument, which can be used to assess the side effects associated with dexamethasone. These side effects may outweigh the benefits of dexamethasone when used with moderately emetogenic chemotherapy.

## Figures and Tables

**Table 1:** Dexamethasone symptom questionnaire

**Dexamethasone questionnaire**		**Patient number**:		□	
Patient Initials:					
	□□□				
Date of birth (day, month, year):					
	□□□				
Today's date (day, month, year):					
	□□□				
We are interested in some things about you and your health. Please answer all the questions by circling the number that best applies to you. There are no “right” or “wrong” answers. The information that you provide will remain strictly confidential.					
**DURING THE WEEK AFTER YOUR CHEMOTHERAPY**:					
		Not at all	A little	Quite a bit	Very much
1.	Did you have indigestion/heartburn/reflux or discomfort in the upper abdomen?	1	2	3	4
2.	Did you have trouble getting to sleep?	1	2	3	4
3.	Have you felt nauseated?	1	2	3	4
4.	Have you vomited?	1	2	3	4
5.	Have you lacked appetite?	1	2	3	4
6.	Have you had increased appetite?	1	2	3	4
7.	Have you had hiccups?	1	2	3	4
8.	Have you lost weight?	1	2	3	4
9.	Have you gained weight?	1	2	3	4
10.	Have you felt agitated/nervous?	1	2	3	4
11.	Have you had a rash/acne on your face?	1	2	3	4
12.	Have you had thrush/yeast infection in your mouth?	1	2	3	4
13.	Did you experience feelings of depression on stopping the dexamethasone?	1	2	3	4

**Table 2:** Patient characteristics

	**Number (%)**
Total	60
Male/female	14(23)/46(77)
	
*Age (years)*	
Mean/median	53/53.5
Range	28–78
	
*Diagnosis*	
Breast cancer	32 (53)
Colorectal cancer	16 (27)
Gynaecological cancer	6 (10)
Lung	2 (3)
Prostate	1 (2)
Unknown primary	3 (5)
	
*Treatment*	
Adjuvant	30 (50)
Metastatic	30 (50)
	
*Smoking status*	
Non-smoker	31 (52)
Ex-smoker	20 (33)
Smoker	8 (13)
Not available	1 (2)
	
*Alcohol consumption*	
Nil	39 (65)
⩽100 g/week	13 (22)
>100 g/week	5 (8)
Not available	3 (5)
	
*Chemotherapy regimen* a	
AC±T	14 (23)
CEF	13 (22)
FOLFOX	8 (13)
XELIRI	3 (5)
Other	16 (27)

a AC±T=Adriamycin and cyclophosphamide± → taxane; CEF=cyclophosphamide, epirubicin, 5-fluorouracil; FOLFOX=oxaliplatin, 5-fluorouracil and folinic acid; XELIRI=capecitabine (Xeloda) and irinotecan.

**Table 3:** Symptoms reported during the week after chemotherapy in patients receiving oral dexamethasone (*n*=60)

	**Percent affected**			
**Symptom**	**Not at all**	**A little**	**Quite a bit**	**A lot**
Insomnia	27	28	20	25
Indigestion/reflux/epigastric discomfort	35	38	15	12
Agitation	43	30	20	7
Increased appetite	50	32	12	7
Weight gain	60	23	13	3
Facial rash/acne	68	17	10	5
Depression on ceasing dexamethasone	75	18	3	3
Hiccups	75	17	8	0
Oral candida	87	10	2	2
Anorexia	45	23	28	3
Nausea	25	47	21	7
Vomiting	80	12	3	5

**Table 4:** Correlation between DSQ items: spearman rank correlation (*P*-value)

	**Epigastric discomfort**	**Insomnia**	**↑ Appetite**	**Hiccups**	**↑ Weight**	**Agitation**	**Acne**	**Thrush**	**Depression**	**Nausea**
Insomnia	0.26*									
	0.048									
Increased appetite	0.16	0.05								
	0.23	0.71								
Hiccups	−0.07	0.06	0.00							
	0.57	0.63	0.99							
Increased weight	0.12	0.21	0.50*	−0.08						
	0.36	0.100	<0.001	0.56						
Agitation	0.26*	0.27*	0.10	0.01	−0.02					
	0.044	0.040	0.45	0.93	0.85					
Acne	0.05	0.10	0.20	−0.14	0.17	0.06				
	0.69	0.47	0.13	0.29	0.19	0.65				
Thrush	0.05	0.37*	−0.03	0.34*	−0.13	0.22	0.11			
	0.73	0.004	0.83	0.008	0.31	0.096	0.38			
Depression	0.28*	0.14	−0.02	−0.18	0.09	0.55*	0.05	0.01		
	0.029	0.28	0.89	0.17	0.48	<0.001	0.72	0.95		
Nausea	0.28*	−0.02	−0.23	−0.04	−0.04	0.26*	0.04	0.15	0.31*	
	0.033	0.87	0.074	0.79	0.73	0.046	0.74	0.24	0.016	
Vomiting	0.03	0.20	−0.28*	−0.00	−0.07	0.12	0.10	0.19	0.14	0.38*
	0.82	0.12	0.031	0.98	0.59	0.37	0.47	0.15	0.29	0.003

DSQ=Dexamethasone Symptom Questionnaire.

* Statistically significant (*P*-value<0.05).
